# Contacts with general practitioners, dentists, and medical specialists among nursing home residents: a cross-sectional study in 44 German nursing homes

**DOI:** 10.1186/s12913-021-07429-6

**Published:** 2022-01-06

**Authors:** Jonas Czwikla, Annika Schmidt, Maike Schulz, Ansgar Gerhardus, Guido Schmiemann, Karin Wolf-Ostermann, Daniel Gand, Anna-Carina Friedrich, Falk Hoffmann, Heinz Rothgang

**Affiliations:** 1 Department of Health, Long-term Care and Pensions, SOCIUM Research Center on Inequality and Social Policy, University of Bremen, Mary-Somerville-Straße 5, 28359 Bremen, Germany; 2 High-Profile Area of Health Sciences, University of Bremen, Bibliothekstraße 1, 28359 Bremen, Germany; 3 Department for Health Care Research, Institute of Public Health and Nursing Research (IPP), University of Bremen, Grazer Straße 4, 28359 Bremen, Germany; 4 Department for Health Services Research, Institute of Public Health and Nursing Research (IPP), University of Bremen, Grazer Straße 4, 28359 Bremen, Germany; 5 Department of Health Services Research, Carl von Ossietzky University of Oldenburg, Ammerländer Heerstraße 114-118, 26129 Oldenburg, Germany

**Keywords:** Medical care, Nursing home, Nursing home resident, General practitioner, Dentist, Medical specialist, Health services research

## Abstract

**Background:**

Nursing home residents have high medical care needs. Their medical care utilization is, however, lower compared to community-dwelling elderly and varies widely among nursing homes. This study quantified the utilization of general practitioners (GPs), dentists, and medical specialists among nursing homes and residents, and investigated whether dentist utilization is associated with individual and nursing home characteristics.

**Methods:**

Forty-four nursing homes invited 2124 residents to participate in a cross-sectional study. For 10 medical specialties, data on contacts in nursing homes, practices, and by telephone in the last 12 months were assessed at individual and nursing home level. The proportion of nursing homes and residents with any form of contact, and the median number and interquartile range (IQR) of contacts among individuals with contact were determined. Using multilevel logistic regression, associations between the probability of individual dental care utilization and sex, age, LTC grade, years of residence, sponsorship, number of nursing home beds, and transport and medical escort services for consultations at a practice were investigated.

**Results:**

The proportion of nursing homes with any form of contact with physicians ranged from 100% for GPs, dentists, and urologists to 76.7% for gynecologists and orthopedists. Among the nursing homes, 442 residents participated (20.8% response). The proportion of residents with any contact varied from 97.8% for GPs, 38.5% for neurologists/psychiatrists, and 32.3% for dentists to 3.0% for gynecologists. Only for GPs, neurologists/psychiatrists, dentists, otorhinolaryngologists, urologists, and dermatologists, the proportion was higher for nursing home contacts than for practice and telephone contacts. Among residents with any contact, the median number of contacts was highest for GPs (11.0 [IQR 7.0-16.0]), urologists (4.0 [IQR 2.0-7.0]), and neurologists/psychiatrists (3.0 [IQR 2.0-5.0]). Dentist utilization varied widely among nursing homes (median odds ratio 2.5) and was associated with higher age.

**Conclusions:**

Almost all residents had regular contact to GPs, but only one third had contact with dentists. Lower proportions with contact were found for medical specialists, except for neurologists/psychiatrists. Reasons for the large variations in dental care utilization among nursing homes should be identified.

**Trial registration:**

DRKS00012383 [2017/12/06].

## Background

Globally, the share of older people is increasing rapidly [1]. Since older individuals suffer more frequently from physical and mental impairments, the number of people in need of long-term care (LTC) is also growing [2]. In Germany, for example, the proportion of people aged 80 years and over is expected to increase from 7.2% in 2020 to 13.0% in 2050 and the number of LTC dependents is estimated to rise from 4.3 million to 6.5 million [3]. Twenty percent of all LTC dependents in Germany reside in one of currently 11,317 nursing homes [3].

Most nursing home residents suffer from multimorbidity, frailty, cognitive impairments, and polypharmacy, all of which necessitate access to and utilization of medical care [4–7]. In nursing homes, almost all residents have regular contact with general practitioners (GPs) [8–12]. However, only approximately half of all residents have contact with dentists at least once a year [8, 10, 11]. In previous German studies, dental care utilization varied widely among nursing homes [10, 11], and some homes even reported no regular dental contacts for any of their residents [13]. This is surprising, since nursing home residents usually have high dental care needs resulting from poor oral health, which is negatively associated with quality of life [14–17]. Moreover, it has been shown that, with the exception of neurologists and psychiatrists, the proportion of individuals having contact with medical specialists at least once per year is lower among nursing home residents than among community-dwelling elderly [4, 5], and contact rates also vary widely among nursing homes [10, 11]. However, the influence of nursing home characteristics has not been studied in detail so far.

Given the low proportion of nursing home residents having regular contact with dentists and medical specialists, it is often claimed that a significant proportion of residents does not receive appropriate medical care [10, 11, 18–20]. However, in order to draw a complete picture of medical care provision in this setting, it is also necessary to know the number of consultations (i.e., the intensity of medical care) and whether these take place in the nursing home, at a practice, or by telephone. All types of contacts can be relevant for the medical care process (e.g., telephone contacts with GPs during a pandemic and prior to unplanned hospital stays [21, 22]). However, previous studies focused particularly on proportions of nursing homes and nursing home residents having contact with physicians [8, 10, 11, 13] but did not systematically investigate the number and types of contact. Furthermore, large variations in dental care utilization among nursing homes have been reported, although in Germany at least one dental contact per year is recommended to the entire population and it can therefore be assumed that all nursing home residents are in need of dental care [10, 11]. These variations should be investigated considering both individual and nursing home characteristics.

The purpose of this study was i) to quantify the utilization of GPs, dentists, and medical specialists among nursing homes and nursing home residents, and ii) to investigate whether dental care utilization is associated with individual and nursing home characteristics.

## Methods

### Design and setting

This cross-sectional study was conducted as part of the “Needs-based provision of medical care to nursing home residents” mixed-methods study, which is described in detail elsewhere [23]. In brief, the cross-sectional study was carried out in nursing homes in the German federal states of Bremen and Lower Saxony between February 2018 and March 2019. The recruitment strategy comprised two steps: First, all nursing homes in Bremen (*n* = 87) and a convenience sample of nursing homes in Lower Saxony (*n* = 262) were invited to participate. The 44 nursing homes (12.6% response) that agreed to participate in turn invited all eligible residents (approximately *n* = 2124) or their relatives/legal guardians to participate and provide informed consent. Eligibility criteria were i) LTC dependent, ii) at least 60 years old, and iii) resident of a nursing home for at least 12 months.

In Germany, medical and nursing care are financed either by statutory health and LTC insurance, which covers approximately 90% of the population, or by private health and LTC insurance, covering the remaining 10%. When an application for LTC is submitted, the Medical Advisory Service of the respective insurance assesses the amount of benefits individuals can receive to organize LTC in a nursing home (or in the community). The assessment differentiates 5 LTC grades, whereby Grade 1 is approved for low and Grade 5 for high care needs. Benefit levels are capped, and the remaining costs must be paid out of pocket or by social assistance [24, 25]. Irrespective of whether they live in the community or a nursing home, all individuals may freely choose their GPs, dentists, and medical specialists, and costs are covered by their respective insurance.

### Standardized assessment of nursing homes

A questionnaire was completed by the management of the participating nursing homes to assess data on the characteristics of the homes (i.e., sponsorship, number of nursing home beds, cooperation agreements with physicians [i.e., commitments to regular contact and increased remuneration], and transport and medical escort services for contacts at a practice) [23]. The administrative employees were also asked whether at least one resident of the nursing homes had contact with GPs, dentists, urologists, neurologists/psychiatrists, otorhinolaryngologists, ophthalmologists, surgeons, dermatologists, gynecologists, and orthopedists in the last twelve months. This question was asked separately for consultations at a medical practice, in a nursing home, and by telephone.

### Standardized assessment of nursing home residents

A standardized assessment of the participating nursing home residents was conducted by trained study nurses. This included, inter alia, a review of nursing records to obtain information on sex, age, LTC grade, and years of residence in the nursing home [23]. Data on the number of contacts with GPs, dentists, and medical specialists in the last twelve months, differentiating between contacts at a practice, in the nursing home, and by telephone, were also obtained from the nursing records.

In addition, a questionnaire on the utilization of medical care was completed for each resident by one of the residents’ care nurses [23]. The care nurses were asked whether the residents’ relatives take care of the residents’ GP and medical specialist utilization. Response options were “yes, organization”, “yes, as a companion”, “no”, and “unknown” (multiple answers possible). Further questions related to the main organizers of GP and medical specialist utilization in the last twelve months.

### Statistical analysis

First, the characteristics of the participating nursing homes and residents were analyzed. Among the nursing homes, the distribution of sponsorship (independent nonprofit, private, public/municipal), the mean number of nursing home beds, the proportions of nursing homes having cooperation agreements with GPs and dentists, as well as the proportions of nursing homes providing transport and medical escort services were examined. For the residents, the distributions by sex (female, male), age group (60-74, 75-84, 85+ years), and LTC grade (1/2, 3, 4/5) as well as the mean age and the mean years of residence were determined. Furthermore, the proportions of individuals whose relatives take care of GP and medical specialist utilization were calculated.

Second, the proportions of residents whose GP utilization and medical specialist utilization were primarily organized by nursing home staff, the nursing home resident, a relative/legal guardian, a GP (only applicable to GP utilization), and a medical specialist (only applicable to medical specialist utilization) were determined.

Third, the proportions of nursing homes and residents with at least one contact with GPs, dentists, and medical specialists in the last twelve months were calculated. The analyses were also conducted separately for contacts at a practice, in the nursing home, and by telephone. Furthermore, the mean and median numbers as well as the standard deviation (SD) and interquartile range (IQR) of contacts among residents with at least one contact were determined.

Finally, a multilevel logistic regression analysis with random intercepts only was conducted to investigate whether the probability of individual dental care utilization in the last twelve months is associated with individual and nursing home characteristics. The multilevel regression with a nursing home clustering variable was conducted i) without any explanatory variables, ii) including only the individual variables sex, age group, LTC grade, and years of residence in quartiles as fixed effects, and iii) including additionally the nursing home variables sponsorship, number of nursing home beds in quartiles, and having or not having transport and medical escort services as fixed effects. Because the individual-level variable *relatives taking or not taking care of medical specialist utilization* and the nursing home-level variable *having or not having cooperation agreements with dentists* had large proportions of missings and were not associated with the outcome in preceding analyses, these variables were not considered in the final multilevel regression. The variance inflation factor (VIF) with a cutoff value of 10 was used to assess multicollinearity between the explanatory variables [26]. The median odds ratio (MOR) was considered as measure of variation [27, 28]. The MOR indicates the MOR one nursing home resident would have when moving between two randomly chosen nursing homes to the nursing home with a higher probability of utilizing dental care [27]. For example, a MOR of 1.0 would indicate that there is no nursing home-level variance in dental care utilization, whereas a MOR greater than 1.0 would indicate that there is nursing home-level variation [27, 28]. Model fit was assessed using the likelihood ratio test [29].

All analyses were conducted using SAS 9.4 (SAS Institute Inc., Cary, NC, USA). For the multilevel analysis, PROC GLIMMIX [29] was used.

## Results

### Nursing home and nursing home residents’ characteristics

Of the residents eligible for the study, 442 (20.8% response) provided informed consent. The analysis included 43 (97.7%) of 44 participating nursing homes and 400 (90.5%) of 442 nursing home residents. One nursing home which gave no information on its characteristics and 42 residents with invalid information on age (*n* = 8), LTC grade (*n* = 7), or years of residence (*n* = 27) were not considered.

Among the nursing homes, 51.2% were independent nonprofit nursing homes, 43.9% were privately sponsored, and 4.9% were public/municipal nursing homes (Table 1). The mean number of nursing home beds was 87.2 (SD 48.8). Forty percent of the nursing homes had cooperation agreements with GPs and 60% with dentists. Extra services for visits to medical practices were available in 23.3% (transport service) and 58.5% (medical escort service) of the nursing homes.

Among the nursing home residents, one third were men. The mean age was 83.0 years (SD 9.4) and more than 80% of all residents had a LTC grade of 3 or higher. The mean time of residence was 4.4 years (SD 4.3). Relatives took care of medical care utilization for 26.3% (GP utilization) and 34.1% (medical specialist utilization) of the residents.

**Table 1:** Characteristics of the participating nursing homes and nursing home residents

Category	%
*Nursing home characteristics*	
**Sponsorship (*n*** **= 41)**	
Independent nonprofit	51.2
Private	43.9
Public/municipal	4.9
**Number of nursing home beds (*n*** **= 43)**	
Mean (SD); Median (IQR)	87.2 (48.8); 77.0 (58.0-93.0)
**Cooperation agreements with GPs (n = 40)**	
Yes	40.0
**Cooperation agreements with dentists (n = 40)**	
Yes	60.0
**Transport service for contacts at a practice (n = 43)**	
Yes	23.3
**Medical escort service for contacts at a practice (n = 41)**	
Yes	58.5
*Nursing home residents’ characteristics*	
**Sex (*n*** **= 400)**	
Male	33.3
**Age group (n = 400)**	
60-74 years	18.8
75-84 years	33.5
85+ years	47.8
Mean (SD); Median (IQR)	83.0 (9.4); 84.0 (77.0-90.0)
**Long-term care grade (n = 400)**	
1/2	19.8
3	37.8
4/5	42.5
**Years of residence (n = 400)**	
Mean (SD)	4.4 (4.3); 3.0 (1.8-5.5)
**Relatives take care of GP utilization (*n*** **= 365)**	
Yes	26.3
**Relatives take care of medical specialist utilization (*n*** **= 364)**	
Yes	34.1

*Abbreviations*: *SD* standard deviation, *IQR* interquartile range, *GP* general practitioner

### Organizers of medical care utilization

Both GP and medical specialist utilization for the participating residents were primarily organized by nursing home staff (Table 2). GPs were involved more frequently as a main organizer in the organization of medical care than residents, relatives/legal guardians, and medical specialists.

**Table 2:** Main organizers of general practitioner and medical specialist utilization among the participating nursing home residents (multiple answers possible)

Main organizers	Nursing home residents (***n*** = 400)	
	general practitioner utilization	medical specialist utilization^**a**^
	%	%
Nursing home staff	94.3	93.2
Nursing home resident	5.8	5.6
Relative/legal guardian	5.0	8.6
General practitioner	16.8	N/A
Medical specialists	N/A	8.1

*Abbreviation*: *N/A* not applicable

^a^ Nursing home residents without medical specialist utilization were not included (*n* = 5)

### Contacts with physicians

All nursing homes had contact with GPs at least once in the last twelve months (Table 3). The proportion with at least one contact was highest for contacts in the nursing home (100%), followed by practice (86.1%) and telephone (83.7%) contacts. Among nursing home residents, the proportion having any contact with GPs was 97.8%. It was highest for contacts in the nursing home (93.8%), followed by telephone (74.5%) and practice (18.8%) contacts. Among residents with any contact to GPs, the mean and median numbers of contacts were 12.5 (SD 9.2) and 11.0 (IQR 7.0-16.0), respectively. They were highest for contacts in the nursing home, followed by telephone and practice contacts.

**Table 3:** Contacts to general practitioners, dentists, and medical specialists among the participating nursing homes and nursing home residents

Medical specialty	Nursing homes (***n*** = 43)	Nursing home residents (***n*** = 400)	
	Proportion with at least one contact in the last twelve months	Proportion with at least one contact in the last twelve months	Number of contacts among those with any contact in the last twelve months
	%	%	Mean (SD); Median (IQR)
**General practitioners**			
Any contact	100.0	97.8	12.5 (9.2); 11.0 (7.0-16.0)
Contact in a practice	86.1	18.8	0.5 (1.8); 0.0 (0.0-0.0)
Contact in the nursing home	100.0	93.8	7.1 (6.5); 6.0 (1.0-7.0)
Contact by telephone	83.7	74.5	4.9 (6.5); 3.0 (1.0-7.0)
**Dentists**			
Any contact	100.0	32.3	2.1 (1.6); 1.0 (1.0-2.0)
Contact in a practice	86.1	15.5	0.9 (1.4); 0.0 (0.0-1.0)
Contact in the nursing home	76.7	21.0	0.9 (1.1); 1.0 (0.0-1.0)
Contact by telephone	32.6	5.3	0.2 (0.6); 0.0 (0.0-0.0)
**Urologists**			
Any contact	100.0	18.0	5.1 (4.7); 4.0 (2.0-7.0)
Contact in a practice	83.7	7.5	0.8 (1.5); 0.0 (0.0-1.0)
Contact in the nursing home	67.4	10.8	2.9 (3.4); 2.0 (0.0-5.0)
Contact by telephone	44.2	8.0	1.4 (3.0); 0.0 (0.0-2.0)
**Neurologists/psychiatrists**			
Any contact	97.7	38.5	3.7 (3.0); 3.0 (2.0-5.0)
Contact in a practice	65.1	5.3	0.2 (0.8); 0.0 (0.0-0.0)
Contact in the nursing home	86.1	33.0	2.6 (2.5); 2.0 (1.0-3.0)
Contact by telephone	55.8	14.5	0.8 (1.7); 0.0 (0.0-1.0)
**Otorhinolaryngologists**			
Any contact	95.4	21.8	1.8 (1.3); 1.0 (1.0-2.0)
Contact in a practice	76.7	7.0	0.6 (1.4); 0.0 (0.0-1.0)
Contact in the nursing home	46.5	15.0	1.0 (0.9); 1.0 (0.0-2.0)
Contact by telephone	32.6	2.5	0.2 (0.5); 0.0 (0.0-0.0)
**Ophthalmologists**			
Any contact	88.4	17.3	2.3 (1.9); 2.0 (1.0-3.0)
Contact in a practice	86.1	14.0	1.6 (1.8); 1.0 (1.0-2.0)
Contact in the nursing home	9.3	3.5	0.4 (0.9); 0.0 (0.0-0.0)
Contact by telephone	30.2	3.5	0.3 (0.7); 0.0 (0.0-0.0)
**Surgeons**			
Any contact	88.4	6.8	2.1 (1.7); 2.0 (1.0-3.0)
Contact in a practice	88.4	5.0	1.1 (0.9); 1.0 (0.0-2.0)
Contact in the nursing home	4.7	2.0	0.4 (0.8); 0.0 (0.0-1.0)
Contact by telephone	20.9	1.5	0.5 (1.3); 0.0 (0.0-0.0)
**Dermatologists**			
Any contact	86.1	17.0	3.3 (4.5); 2.0 (1.0-4.0)
Contact in a practice	83.7	5.3	0.6 (1.0); 0.0 (0.0-1.0)
Contact in the nursing home	41.9	11.8	1.6 (2.0); 1.0 (0.0-2.0)
Contact by telephone	39.5	6.5	1.1 (2.6); 0.0 (0.0-1.0)
**Gynecologists**			
Any contact	76.7	3.0^a^	2.0 (1.4); 1.5 (1.0-2.5)
Contact in a practice	74.4	1.5^a^	0.5 (0.5); 0.5 (0.0-1.0)
Contact in the nursing home	18.6	0.8^a^	0.4 (0.7); 0.0 (0.0-0.5)
Contact by telephone	20.9	1.5^a^	1.1 (1.4); 0.5 (0.0-2.5)
**Orthopedists**			
Any contact	76.7	5.5	2.2 (1.4); 2.0 (1.0-3.0)
Contact in a practice	69.8	4.5	1.5 (1.3); 1.0 (1.0-2.0)
Contact in the nursing home	9.3	1.3	0.4 (0.9); 0.0 (0.0-0.0)
Contact by telephone	16.3	1.3	0.4 (0.9); 0.0 (0.0-0.0)

*Abbreviations*: *SD* standard deviation, *IQR* interquartile range

^a^ *n* = 267 female nursing home residents

All nursing homes also had contact with dentists at least once. The proportion was highest for contacts at a practice (86.1%), followed by nursing home (76.7%) and telephone (32.6%) contacts. Among the residents, one third had at least one contact with a dentist. The proportion was highest for nursing home contacts (21.0%), followed by contacts at a practice (15.5%) and by telephone (5.3%). The mean and median numbers of contacts among individuals with any contact were 2.1 (SD 1.6) and 1.0 (IQR 1.0-2.0). They were nearly identical for nursing home and practice contacts, but lower for telephone contacts.

Regarding other medical specialties, the proportion of nursing homes with at least one contact ranged from 100% for urologists to 76.7% for gynecologists and orthopedists. As with GPs, the proportion with contact at least once with neurologists/psychiatrists was highest for contacts in the nursing home, whereas for all other medical specialties the proportion was highest for contacts at a practice. Among residents, the proportion with any form of contact ranged from 38.5% for neurologists/psychiatrists to 5.5% for orthopedists and 3.0% for gynecologists. Whereas for neurologists/psychiatrists, urologists, otorhinolaryngologists, and dermatologists, the proportion with at least one contact was highest for contacts in the nursing home, for ophthalmologists, surgeons, and orthopedists, it was highest for contacts at a practice. For gynecologists, the proportion was identical for practice and telephone contacts. Among residents having any contact with a specific medical specialist, the highest total mean and median numbers of contacts were observed for urologists (mean 5.1 [SD 4.7]; median 4.0 [IQR 2.0-7.0]) and neurologists/psychiatrists (mean 3.7 [SD 3.0]; median 3.0 [IQR 2.0-5.0]). Among these medical specialties as well as among otorhinolaryngologists and dermatologists, most contacts took place in a nursing home. Among ophthalmologists, surgeons, and orthopedists, most contacts were contacts at a practice, whereas among gynecologists, telephone contacts were most common.

### Multilevel analysis

In the multilevel logistic regression analysis (*n* = 375), the MOR of 2.37 in the empty model indicated large nursing home-level differences in dental care utilization (Table 4). The inclusion of individual level-variables increased the MOR to 2.68. Compared to the empty model, the proportional change in nursing home-level variance was + 29.8%. In the full model with individual and nursing home-level variables, the MOR was 2.47, and the proportional change in variance was − 15.5% compared to the previous model. With the exception of a positive association between higher age and dental care utilization, no significant associations were determined. The VIF ranged from 1.20 to 2.34 and indicated no multicollinearity.

**Table 4:** Multilevel logistic regression analysis for the probability of individual dental care utilization in the last twelve months (*n* = 375)

	Model 1: empty model		Model 2: +individual predictors		Model 3: +contextual predictors	
			OR	95% CI	OR	95% CI
*Individual-level variables*						
**Sex (ref. female)**						
Male			0.86	(0.49-1.50)	0.88	(0.50-1.54)
**Age group (ref. 60-74 years)**						
75-84 years			1.76	(0.83-3.75)	1.87	(0.87-4.00)
85+ years			**2.27**	**(1.03-4.98)**	**2.43**	**(1.10-5.38)**
**Long-term care grade (ref.** **1/2**)						
3			1.31	(0.64-2.66)	1.30	(0.64-2.65)
4/5			0.98	(0.48-1.98)	0.99	(0.49-2.02)
**Years of residence (ref. quartile 1)**						
Quartile 2			0.90	(0.45-1.79)	0.82	(0.41-1.64)
Quartile 3			0.95	(0.47-1.93)	0.88	(0.43-1.80)
Quartile 4			0.76	(0.36-1.59)	0.70	(0.33-1.47)
*Nursing home-level variables*						
**Sponsorship (ref. independent nonprofit)**						
Private					0.85	(0.34-2.08)
Public/municipal					2.31	(0.36-15.07)
**Number of nursing home beds (ref. quartile 1)**						
Quartile 2					1.01	(0.30-3.37)
Quartile 3					0.95	(0.25-3.68)
Quartile 4					2.50	(0.68-9.22)
**Transport service for contacts at a practice (ref. no)**						
Yes					1.45	(0.55-3.85)
**Medical escort service for contacts at a practice (ref. no)**						
Yes					1.27	(0.50-3.23)
*Mea’sures of variation*						
**Nursing home-level variance (SE)**	0.822 (0.392)		1.066 (0.489)		0.901 (0.426)	
**Proportional change in variance**			+ 29.8%		−15.5%	
**MOR**	2.37		2.68		2.47	
*Fit statistics*						
**–2 log likelihood**	461.6		453.6^a^		448.4^a^	

*Abbreviations*: *OR* odds ratio, *CI* confidence interval, *ref* reference, *SE* standard error, *MOR* median odds ratio

Boldface indicates statistical significance

^a^No significant likelihood ratio test

This analysis included only 375 nursing home residents because for 25 residents, no complete information was available for the nursing home-level variables

## Discussion

This study quantified the utilization of GPs, dentists, and medical specialists among nursing homes and residents, and investigated whether dental care utilization is associated with individual and nursing home characteristics. We found that all nursing homes and almost all residents had regular contact with GPs in the last twelve months, and nursing home and telephone contacts were the most relevant contact types. Moreover, all nursing homes, but only one third of the residents had contact with dentists at least once. Except for neurologists/psychiatrists, lower proportions with at least one contact were found for all other medical specialties. Medical care most frequently took place in the nursing home, and GPs were the medical specialty with the highest proportion of telephone contacts. Dentist utilization varied widely among nursing homes and was positively associated with higher age. For nursing home characteristics, no associations were found.

Regarding the utilization of medical care, our study shows that contacts with GPs in the nursing home and by telephone are the most common contact types. On average, nursing homes are visited by 8.6 different GPs [13] and, according to our study, residents have more than seven contacts with GPs in the nursing home, indicating a high prevalence of need for general medical care. Although treatment options by phone (e.g., between GPs and nursing home residents or the residents’ relatives or care nurses) are limited, our study further shows that residents have almost five telephone contacts with GPs per year. Given this finding, and given the expected importance of telephone contacts during a pandemic and in emergency cases [21, 22], the importance of telephone contacts is probably greater than expected. Future studies should therefore systematically investigate the role of telephone contacts in the provision of medical care in nursing homes. Particularly, they should identify the underlying reasons for, and consequences of, telephone contacts as well as the proportion of telephone contacts supporting and substituting other types of contact.

With respect to the utilization of dentists, our study demonstrates that all nursing homes have contact with dentists, but only 77% are visited by dentists. This is comparable to Schröder et al. [13], who found that only 85% of nursing homes are regularly visited by dentists. In our study, we further showed that nursing home residents utilize dental care most frequently in the nursing home and at dental practices. However, we also found that only 32% of the residents had at least one contact, which is even lower than the 40-55% reported previously [8, 10, 11]. Although oral health among nursing home residents might be better than among home care recipients [30], it is expected that there is a large disparity between dental care needs and utilization among nursing home residents [11, 18, 19, 31–33]. This is especially true considering that in Germany at least one contact with a dentist per year is recommended to the entire population and individuals who follow this advice receive additional insurance benefits if they need dentures or crowns [34]. As our study shows, dental care utilization varies widely between nursing homes. These variations could not be explained by the factors examined in this study. Future studies should therefore systematically investigate the wide variations among nursing homes and identify examples of best practice. This could provide useful information for the design of future interventions that would be most effective for improving dental care provision in nursing homes. Furthermore, the implementation of tools for the assessment of dental care needs and clear pathways for the organization of dental care in the nursing home and, where necessary, at a practice, could help to increase dental care utilization [35–37].

Regarding the utilization of medical specialists, our study shows that almost all nursing homes have contact with all medical specialties. Looking only at the contacts in nursing homes, our proportions with contact are comparable to those reported by Schröder et al. [13]: neurologists/psychiatrists (86% vs. 90%), urologists (67% vs. 57%), otorhinolaryngologists (47% vs. 39%), and ophthalmologists (9% vs. 18%). Among the residents, our study demonstrates that neurologists/psychiatrists, urologists, otorhinolaryngologists, and dermatologists are more likely to provide medical care in the nursing home than at a practice, whereas physicians such as ophthalmologists, surgeons, and orthopedists, who require more unwieldy technical equipment for the provision of medical care, are more likely to provide medical care at their practice. This finding is relevant for the medical care process because nursing home residents often require transport and a medical escort to appointments at a practice. To improve the situation, strong cooperation between GPs, care nurses, medical specialists, and relatives is essential [38–40]. Spreckelsen et al. [8], however, found that the coordination of medical specialist care by GPs deteriorates after nursing home admission. Assessment tools and clear pathways for the organization of medical specialist care could therefore help to assess whether and how often specialist contacts in the nursing home, a practice, or by telephone are required, and to optimize the provision of medical specialist care to nursing home residents [41, 42].

### Strengths and limitations

A major strength of this study is the large number of participating nursing homes, allowing us to investigate heterogeneity in the utilization of medical care among nursing homes. Furthermore, to our knowledge, this is the first study that investigated not only the proportion of nursing homes and residents having contact with physicians, but also the number and type of contacts. Another strength is that data on both nursing homes and their residents were analyzed.

There are, however, some important limitations to consider. First, a claims data-based non-response analysis revealed that the participating nursing home residents were younger, had fewer contacts with GPs, and were less likely to die within the next twelve months compared to the nonparticipants [43]. Due to this non-response bias, the generalizability of our results is limited, and it cannot be ruled out that we underestimated the actual medical care utilization among nursing home residents. Second, only individuals residing in a nursing home for at least 12 months were included, that is, residents who died within the first 12 months after moving into a nursing home were not considered. Since these residents might have different amounts of medical care utilization, our findings cannot be extrapolated to this population group. Third, the number of included residents per nursing home was low. Nevertheless, our multilevel analyses enabled us to assess variations in the utilization of dental care among nursing homes. Fourth, our questionnaires were only pretested in one nursing home with administrative employees, study nurses, and care nurses. The pretest resulted in no changes of the questionnaires. Fifth, our assessment method is susceptible to recall bias. Even though nursing records were reviewed, they might not always be complete. Sixth, the utilization of medical care among nursing home residents was not analyzed in relation to individual medical care needs. For determining valid information on medical specialist care needs, detailed medical assessments are required which were not conducted for all medical specialties and residents. We were therefore unable to determine whether the medical care provision was appropriate. Seventh, there was no information available on whether the utilization of medical specialists was coordinated by GPs, which limited the validity of our results regarding the organization of medical care. Finally, we were unable to consider information on whether or not nursing homes have a person responsible for the organization of dental care as a nursing home-level characteristic and oral health status, comorbidity, limitations in activities of daily living, and cognitive impairments as individual-level characteristics in the multilevel analysis. LTC grades, however, served as a proxy for the missing information on the latter three characteristics.

### Conclusions

Almost all nursing home residents had regular contact with GPs, but only one third had contact with dentists at least once. With the exception of neurologists/psychiatrists, lower proportions with at least one contact were found for all other medical specialties. Future studies should investigate the role of telephone contacts for the provision of medical care in nursing homes. Assessment tools and clear pathways should be developed in order to evaluate whether consultations with specialists are required. Furthermore, reasons for the large variations in dental care utilization among nursing homes should be identified. This could inform the design of future interventions to increase the low dentist utilization and improve the organization of medical care among nursing home residents.

## Data Availability

The datasets generated and/or analyzed during the current study are not publicly available due to data protection, but data sharing is feasible upon reasonable request and in collaboration with the authors and Competence Center for Clinical Trials of the University of Bremen.

## Abbreviations

GP: General practitioner
IQR: Interquartile Range
LTC: Long-term care
MOR: Median odds ratio
SD: Standard deviation
VIF: Variance inflation factor
